# Embryonic Benzo[a]pyrene Exposure Induces Multigenerational Reproductive Effects on Adult Male Medaka: Phenotypic and Transcriptomic Insights

**DOI:** 10.3390/toxics13100886

**Published:** 2025-10-17

**Authors:** Yinhua Chen, Yi Yang, Xian Qin, Jiangang Wang, Guanglong Tang, Rim EL Amouri, Jiayang Chen, Jack Chi-Ho Ip, Wenhua Liu, Jiezhang Mo

**Affiliations:** 1 Guangdong Provincial Key Laboratory of Marine Disaster Prediction and Prevention, Shantou University, Shantou 515063, China; 19yhchen4@stu.edu.cn (Y.C.); 23jgwang@stu.edu.cn (J.W.); 25gltang@stu.edu.cn (G.T.); rimelamouri@stu.edu.cn (R.E.A.); whliu@stu.edu.cn (W.L.); 2 Department of Infectious Diseases and Public Health, City University of Hong Kong, Hong Kong SAR, China; yyang897@cityu.edu.hk; 3 State Key Laboratory of Marine Environmental Health, City University of Hong Kong, Hong Kong SAR, China; xianqin2@cityu.edu.hk; 4 Central Laboratory, Shantou University, Shantou 515063, China; chenjy@stu.edu.cn; 5 Science Unite, Lingnan University, Hong Kong SAR, China; jackip@ln.edu.hk; 6 International Joint Research Center for Marine Ecological Protection and Disaster Prevention, Shantou University, Shantou 515063, China

**Keywords:** emerging contaminants, fish, reprotoxicity, transcriptome, sperm motility, biomarkers

## Abstract

Benzo[a]pyrene (B[a]P), a ubiquitous environmental pollutant, poses a significant threat to male reproductive health, but the underlying latent molecular mechanisms remain virtually unknown. This study investigated the effects of embryonic B[a]P exposure on testicular function and spermatogenesis in F0 and F1 adult male medaka (*Oryzias latipes*). Embryos were exposed to sublethal concentrations (2.5, 20, and 80 μg/L) for 8 days and then raised in clean water until they reached adulthood. Transcriptomic analysis of F0 testicular tissues revealed widespread dysregulation of critical pathways. Exposure impaired the brain–pituitary–gonadal axis by disrupting GnRH signaling and downregulating genes encoding key steroidogenic enzymes (*CYP17A1*, *HSD3B2*), indicating suppressed testosterone biosynthesis. Concurrently, pathways essential for cellular energy metabolism (AMPK signaling, insulin signaling), amino acid biosynthesis, and cytoskeletal organization (actin cytoskeleton, focal adhesion) were profoundly altered. Furthermore, B[a]P activated apoptotic pathways and disrupted the balance between cell survival (PI3K-Akt signaling) and death, compromising spermatogenic cell fate. These molecular disruptions manifested in drastic physiological impairments, including a reduced gonadosomatic index, decreased sperm motility, and compromised fertilization success in F0 males, although these effects were recovered in the F1 generation. This study provides a comprehensive molecular basis for the long-term reproductive toxicity of early-life B[a]P exposure.

## 1. Introduction

Benzo[a]pyrene (B[a]P), mainly generated through the incomplete combustion of organic matters, is one of the principal representatives of polycyclic aromatic hydrocarbons. Owing to its environmental ubiquitousness and persistence, B[a]P has been frequently detected in various environmental matrixes, including air, water, sediments, and soil [1]. In surface waters on a global scale, B[a]P has been detected at concentrations ranging from ng/L to sub-μg/L [1,2]. At some highly polluted spots, the concentrations of B[a]P in surface water were reported to be up to 0.113 mg/L [3] and 7.8 mg/L [4]. Through various exposure routes (e.g., air-, food-, and water-borne), B[a]P presents in animals and exhibits bioaccumulation [5]. This xenobiotic compound can be metabolized into carcinogenic metabolites (e.g., benzo[a]pyrene-7,8-dihydrodiol-9,10-epoxide) by cytochrome P450 within organisms [1,6]. B[a]P metabolites can covalently bind to DNA and thereby induce mutations [7]. Additionally, B[a]P metabolism also induces the excessive production of reactive oxygen spices, leading to oxidative stress [8].

Empirical studies have demonstrated that B[a]P exerts a wide range of effects on animals, including carcinogenicity, developmental toxicity, immunotoxicity, neurotoxicity, and osteotoxicity [8,9,10,11]. B[a]P also exhibits endocrine-disrupting properties and induces adverse reproductive effects including diminished reproductive competence, aberrant reproductive behavioral patterns, disruptions in sexual differentiation, perturbations of physiological and metabolic homeostasis, and dysregulation of molecular signaling pathways [12,13,14,15].

It has been documented in rodent models that exposure to B[a]P at different life stages—such as embryo, juvenile, prepuberty, and adulthood—induces reproductive toxicity, with altered sexual behaviors, reduced testis weights, increased oxidative damage and apoptosis in testicular tissues, elevated mutations in spermatogenic cells, dysregulated steroidogenesis and spermatogenesis, deteriorated sperm quantity and quality, and compromised fertilization capacity in the exposed animals and their offspring [16,17,18,19,20,21,22,23,24,25,26,27,28,29]. In contrast, only several studies have been performed to evaluate the male reproductive health of fish species following B[a]P exposure [12,30,31,32,33]. Specifically, exposure of adult male *Fundulus heteroclitus* to 10 μg/L B[a]P for 28 days showed a dysregulation of fertilization success, associated with reductions in gonadal weight and plasma testosterone level [12]. Histological analysis revealed a nonsignificant increase in spermatogonia cyst size, but the cyst size and ratios of spermatocytes, spermatids, and spermatozoa were not affected; however, this study did not assess the potential morphological and functional changes in sperms [12]. Intraperitoneal administration of the B[a]P at a dose of 50 mg/kg to adult male *Oncorhynchus mykiss* reduced the levels of plasma testosterone and estradiol, possibly in an anti-estrogenic manner [30].

In another study, it was demonstrated that male adult tilapia injected with 3 mg/kg B[a]P every 6 days five times resulted in B[a]P accumulation and consistent reduction in the condition factor, the gonadosomatic index, and the hepatosomatic index. Transcriptomic analysis identified 309 differentially expressed genes (*p* < 0.5 and fold change > ±1), and functional enrichment analysis highlighted that molecular pathways including arachidonic acid metabolism, androgen receptor to prostate-specific antigen signaling, and insulin influence on lipogenesis, pyruvate metabolism, and glycolysis were significantly affected [31]. Notably, Xu and colleagues showed that embryonic exposure to B[a]P at concentrations of 0.126, 1.26, and 12.6 μg/L for 96 h caused reproductive toxicity in adult male zebrafish (*Danio rerio*) [32]. The morphometric changes were associated with a decrease in the percentage of spermatozoa, reductions in fertilization and hatching, downregulation of genes along the brain–pituitary–gonadal axis (e.g., *gnrh3*, *gnrhr3*, *fshβ*, *lhβ*, *lhγ*, *lhrγ*, and *ar*), and hypermethylation of germ cell-specific genes (e.g., *ddx4*, *dnd1*, and *nanos2*) in the testis [32]. These data indicate that embryonic B[a]P exposure compromised the reproductive fitness of adult male fish, but the regulatory network and molecular mechanisms underlying the reproductive impairment of adult male fish induced by embryonic B[a]P exposure are virtually unknown. Furthermore, whether the reproductive toxicity persists in their offspring or is recovered in subsequent generations remains to be determined.

In this study, using medaka (*Oryzias latipes*) as a fish model, it is hypothesized that B[a]P exposure occurs in embryonic stages and causes reproductive impairment in adult males spanning multiple generations through modulating the molecular pathways related to oxidative stress, DNA damage, cell cycle regulation, apoptosis, and spermatogenesis. By integrating morphometric, physiological, and transcriptional data, the novel mechanistic insights underlying reproductive toxicity in adult male medaka fish caused by embryonic B[a]P exposure are presented.

## 2. Materials and Methods

### 2.1. Chemicals and Stock Solution Preparation

Benzo[a]pyrene (B[a]P; CAS No. 57-63-6; purity ≥ 98%) was obtained from Sigma Chemical Co. (St. Louis, MO, USA). A stock solution of B[a]P (200 mg/L) was prepared in HPLC-grade dimethyl sulfoxide (DMSO; Sigma-Aldrich Co.) and stored at −20 °C until it was required.

### 2.2. Medaka Stock Fish Maintenance

Four-month-old sexually mature medaka (*O. latipes*) [34] were housed under conditions approved by the Animal Ethics Committee of Shantou University, China. Fifteen breeding pairs were randomly distributed into 10 tanks (30 × 30 × 30 cm; 20 L) containing filtered tap water. The fish were maintained at 26 ± 0.5 °C on a 14:10 h light/dark cycle and fed *Artemia nauplii* twice daily.

### 2.3. Benzo[a]pyrene Exposure and Multigenerational Experiments

Embryos were collected from stock medaka fish starting from 30 min after the onset of light, in accordance with the standard breeding protocol for medaka [35]. After rinsing, cleaning, and sorting, embryos (30 embryos per replicate, n = 4 replicates for each treatment) were placed in glass Petri dishes (diameter = 7 cm) [36] and filled with 0.4‰ DMSO solution (solvent control) or the B[a]P exposure solution at concentrations of 2.5, 20, and 80 μg/L. Based on our preliminary data and the exposure levels applied in previous studies [12,32], the exposure concentrations of B[a]P in the present study were set at 2.5, 20, and 80 μg/L, which are environmentally realistic. Additionally, no significant difference in embryo mortality was observed between the blank control and solvent control. The B[a]P exposure solutions were collected and analyzed according to the methods described in our previous study [11]. While no B[a]P was detected in the control, the actual exposure concentrations were 2.2 ± 0.4, 17.0 ± 5.0, and 73.0 ± 10.0 μg/L for the 2.5, 20, and 80 μg/L B[a]P treatments, respectively. As our preliminary data show that control embryos start hatching from 9 days post-fertilization when they were incubated at 26 ± 0.5 °C, the embryonic B[a]P exposure lasts for 8 days, with the exposure solution refreshed daily. Starting from day 9, the exposure solutions were replaced with embryonic culture medium, and the freshly hatched larvae were transferred to clean water so that they could grow. After four months, 15 pairs of sexually mature medaka were randomly assigned a 20 L tank filled with clean water for each replicate, and their embryos were collected for breeding F1 adult fish. In this multigenerational study, no B[a]P exposure was executed except during the F0 embryonic period. Determination of fecundity and fertilization in F0 and F1 adult fish (four months old) was conducted, while six-month-old fish were sampled for the assessments of morphometric parameters, sperm velocity, and testicular transcriptomic profiling.

### 2.4. Assessment of Fecundity and Fertilization in Adult Fish

Fecundity and fertilization rates were assessed daily over a 7-day period following the methodology described previously [37]. Embryos were collected daily from each replicate (n = 4 replicates, each with a 5:5 male-to-female ratio) and examined under a stereo microscope (ZEISS Stemi 305, Oberkochen, Germany). Fecundity was expressed as the mean number of eggs produced per female per day. The fertilization rate was calculated as the percentage of fertilized eggs relative to the total eggs collected.

### 2.5. Morphometric Measurement in Adult Fish

At six months of age, ten fish pairs per treatment were dissected for sampling. Morphometric parameters, including Fulton’s condition factor, gonadosomatic index, and hepatosomatic index, were determined according to Qin et al. (2022) [37]. Fulton’s condition factor was calculated as [body weight (g)/length^3^ (cm^3^)], the gonadosomatic index was calculated as [(gonad weight/whole-body weight) × 100], and the hepatosomatic index was calculated as [(liver weight/body weight) × 100].

### 2.6. Sperm Velocity Assessment in Adult Male Fish

A sperm velocity assessment was conducted for the control, 2.5, and 80 μg/L B[a]P treatments in the F0 generation. Semen was collected from ten randomly sampled adult males per treatment. Testes were dissected and placed into 200 μL of filtered artificial seawater. Semen was released by gently squeezing the testes, following the protocol of Qin et al. (2022) [37]. A 15 μL aliquot of the resulting semen solution was immediately transferred to a concave hanging drop slide for analysis. Sperm motility was assessed using the Sperm Motility Analysis System software (Copenhagen Rigshospitalet Image House, version 4.6, Copenhagen, Denmark). For each fish, swimming velocities were recorded across ten individual fields, capturing between 200 and 600 sperm tracks. Analysis was performed from two perspectives: (1) Motion Pattern: Sperm cells were categorized by curvilinear velocity as motile (curvilinear velocity > 25 μm/s), locally motile (curvilinear velocity = 5–25 μm/s), or immotile (curvilinear velocity < 5 μm/s). The percentage of cells in each category was calculated, summing to 100%. The proportion of motile sperm was defined as the progressive motility. (2) Velocity Parameters: The curvilinear velocity, straight-line velocity, and average path velocity were recorded for statistical comparison.

### 2.7. Testicular Transcriptomic Analysis

Testicular transcriptomic analysis was conducted for the control, 2.5, and 80 μg/L B[a]P treatments in F0 generation. Dissected fish testes were pooled (8 testes per pool as a replicate, n = 4 replicates per treatment), and total RNA was extracted from each sample, as described previously [34]. RNA integrity was confirmed with an Agilent 2100 Bioanalyzer (Santa Clara, CA, USA), and only samples with an RIN greater than 8.0 were used for subsequent library preparation. Stranded mRNA sequencing libraries were constructed from 1 µg of total RNA per sample using the TruSeq RNA Library Prep Kit v2 with poly-A selection and sequenced on an Illumina NovaSeq 6000 platform to generate 150 bp paired-end reads, targeting approximately 40 million reads per sample. Raw FASTQ files were first subjected to quality control using FastQC (v0.11.9), and adapter sequences and low-quality bases were trimmed using Trimmomatic (v0.39). High-quality reads (Q > 20) were then aligned to the medaka reference genome (Ensembl ASM223467v1) utilizing the STAR aligner (v2.7.10a) with default parameters for splice-aware alignment, and gene-level counts were generated from the resulting BAM files using featureCounts (part of Subread v2.0.3) against the corresponding genome annotation file. Differential gene expression analysis between experimental conditions was performed in R (v4.3.1) using the DESeq2 package (v1.40.2), with genes considered significantly differentially expressed if they exhibited an adj-*p*-value of < 0.05. Human orthologs of the significant medaka DEGs were identified and subjected to functional enrichment analysis for Gene Ontology (GO) terms and KEGG pathways using the Database for Annotation, Visualization and Integrated Discovery. Transcriptomic raw data were deposited in the NCBI database with an assigned BioProject accession number PRJNA1328451.

### 2.8. Statistical Analysis

Data analysis was performed using GraphPad Prism version 10.4 (GraphPad Software, Boston, MA, USA). Results are presented as mean ± standard error of the mean (SEM) from four independent replicates (n = 4). Statistical comparisons between the control and benzo[a]pyrene treatment groups were conducted using a one-way ANOVA or two-way ANOVA followed by Tukey’s post hoc test or a Chi-square test, where appropriate. A *p*-value of less than 0.05 was considered statistically significant (* *p* < 0.05).

## 3. Results

### 3.1. Embryonic B[a]P Exposure Reduced Survivorship and Hatching Rate

**Figure 1:**
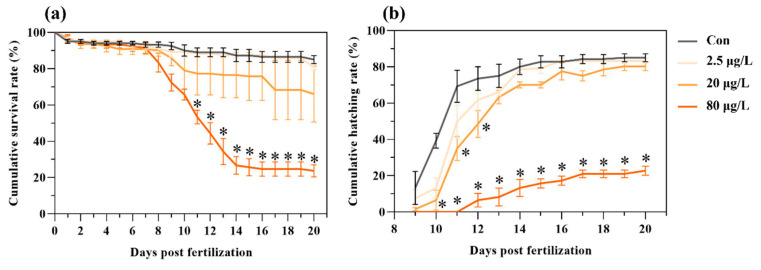
Embryonic exposure to benzo[a]pyrene reduces hatching success and increases mortality in medaka. Dose-dependent effects on (**a**) cumulative survival rate and (**b**) cumulative hatching rate were recorded following exposure to 2.5, 20, and 80 μg/L benzo[a]pyrene. Data represent mean ± SEM (n = 4). * *p* < 0.05 versus control group (Con for control; Chi-square test).

Exposure to B[a]P suppressed development, caused mortality, delayed hatching, and reduced hatching success in medaka embryos (Figure 1). B[a]P caused a dose-dependent embryo mortality during a 20-day exposure period, with a significant increase in mortality starting from 11 days post-fertilization (dpf) in the 80 μg/L B[a]P treatment (Figure 1a). Control embryo started hatching from 9 dpf, but there were 1-day and 2-day delays of first hatching in the 20 and 80 μg/L B[a]P treatments, respectively (Figure 1b). Furthermore, compared to the control, a dose-dependent reduction in hatching success was caused by B[a]P treatments. Notably, the cumulative hatching rate was significantly lower in the 80 μg/L B[a]P treatment starting from 10 dpf and persisting to 20 dpf (Figure 1b).

### 3.2. Embryonic B[a]P Exposure Affected Morphometric Parameters and Reproductive Fitness in the F0 and F1 Adult Fish

**Figure 2:**
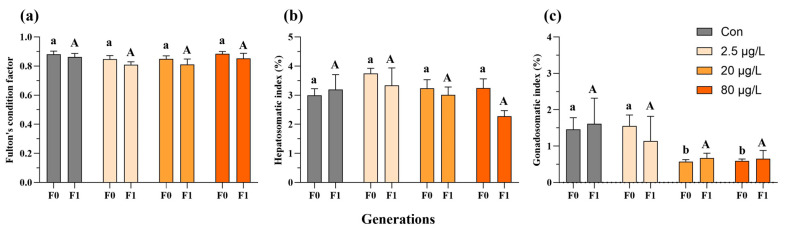
Multigenerational effects of embryonic benzo[a]pyrene exposure on adult male morphometry. Fulton’s condition factor (**a**), hepatosomatic index (**b**), and gonadosomatic index (**c**) of F0 and F1 adult medaka were impacted by an 8-day embryonic exposure to benzo[a]pyrene. Values represent mean ± SEM (n = 4), and statistical analysis was performed using a two-way ANOVA with Tukey’s post hoc test. Different letters are used to show a statistically significant difference within or between benzo[a]pyrene treatments and the corresponding control (lowercase and uppercase letters are used for the F0 and F1 generation, respectively).

**Figure 3:**
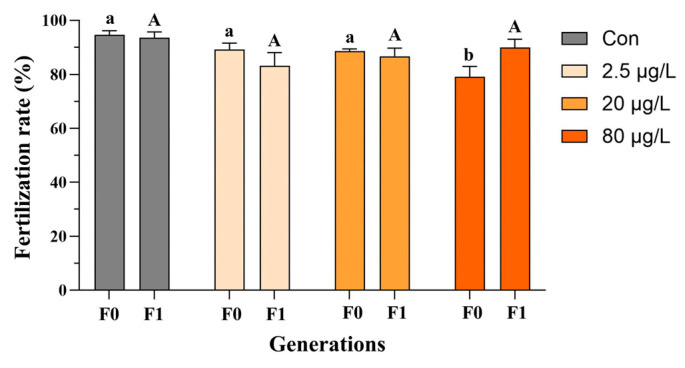
Impaired fertilization in F0 and F1 adult male medaka after embryonic benzo[a]pyrene exposure. Embryos were exposed to benzo[a]pyrene (Con, 2.5, 20, or 80 μg/L) for 8 days. Upon reaching adulthood, the reproductive performance of the directly exposed generation (F0) and their offspring (F1) was evaluated. Data are shown as mean ± SEM (n = 4), and statistical analysis was performed using a two-way ANOVA with Tukey’s post hoc test. Different letters are used to show a statistically significant difference within or between benzo[a]pyrene treatments and the corresponding control (lowercase and uppercase letters are used for the F0 and F1 generation, respectively).

In the F0 generation, embryonic B[a]P exposure did not affect Fulton’s condition factor of adult medaka fish (Figure 2a and Figure S1a), and only the F0 females exhibited a reduction in the hepatosomatic index in the 20 and 80 μg/L B[a]P treatments (Figure S1b) compared to the control. Additionally, decreases in the gonadosomatic index were evident in F0 male fish sampled from the 20 and 80 μg/L B[a]P treatments (Figure 2c). In contrast, none of the above morphometric parameters, namely Fulton’s condition factor, hepatosomatic index, and gonadosomatic index, were significantly affected in both males and females of F1 generation, except an increase in the gonadosomatic index of F1 female fish that descended from embryos exposed to B[a]P at 80 μg/L (Figure 2 and Figure S1). The reproductive fitness of F0-F1 adult fish, namely fecundity and fertilization capability, was assessed as well. Specifically, embryonic B[a]P exposure neither affected the fecundity of F0-F1 adult females nor the fertilization capability of F1 males (Figure 3 and Figure S2). Notably, compared to the control, there was a significant reduction in fertilization rate in the 80 μg/L B[a]P treatment (Figure 3 and Table S1), indicating that embryonic B[a]P exposure for 8 days might impair the fertilization capability of F0 adult males. This might also be attributed to alterations in female gamete quality, oocyte maturation, or oviposition behavior that resulted from the embryonic B[a]P exposure, which remained to be clarified.

### 3.3. Embryonic B[a]P Exposure Affected Sperm Motility of F0 Adult Fish

**Figure 4:**
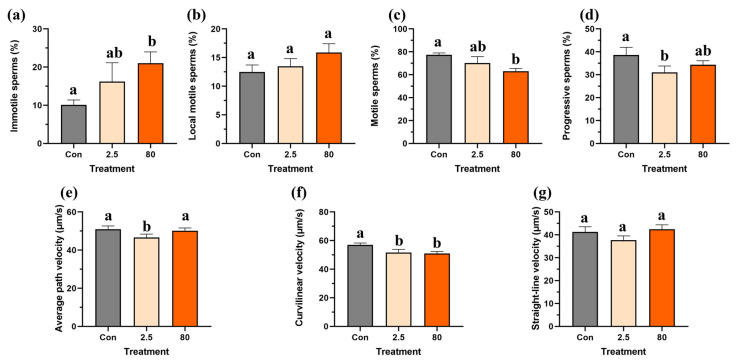
Embryonic benzo[a]pyrene exposure impairs sperm motility in F0 adult male medaka. Spermatozoa from medaka males experiencing an 8-day embryonic benzo[a]pyrene exposure were analyzed for (**a**) immotile, (**b**) local motile, (**c**) motile, and (**d**) progressive sperm populations, as well as for velocity parameters: (**e**) average path velocity, (**f**) curvilinear velocity, and (**g**) straight-line velocity. Data represent mean ± SEM (n = 4), and statistical analysis was performed using a one-way ANOVA with Turkey’s post hoc test. Different letters are used to show a statistically significant difference within or between benzo[a]pyrene treatments and the control (lowercase letters are used for the F0 generation).

For F0 adult male fish, the percentage of immotile and local motile sperm increased in a dose-dependent manner following an 8-day B[a]P exposure (Figure 4a,b), but only the proportion of immotile sperms was significantly elevated in the 80 μg/L B[a]P treatment compared to the control. Additionally, significant reductions in the percentage of motile sperms in the 80 μg/L B[a]P treatment and percentage of progressive sperms in the 2.5 μg/L B[a]P treatment were observed (Figure 4c,d). In contrast, while the straight-line velocity was not affected, another two types of sperm velocity, namely average pathway velocity and curvilinear velocity, were reduced in the F0 adult male fish following an 8-day embryonic B[a]P treatment (Figure 4e–g). Notably, the average pathway velocity significantly decreased in the 2.5 μg/L B[a]P treatment, while the curvilinear velocity was significantly reduced in both 2.5 and 80 μg/L B[a]P treatments (Figure 4e,f) compared to the control.

### 3.4. Embryonic B[a]P Exposure Altered Testicular Transcriptomic Profiles of F0 Adult Fish

The transcriptomic analysis yielded an average of 40.3 million clean reads in F0 adult medaka testicular tissue samples, and the average mapping rate of uniquely mapped reads was 91.0% (Table S2). A total of 71 (including 30 upregulated and 41 downregulated) and 586 (including 347 upregulated and 239 downregulated) DEGs were identified in the 2.5 and 80 μg/L B[a]P treatments with the threshold of adj-*p* < 0.05.

Analyses of GO enrichment (Figures S3 and S4) and KEGG pathways (Table 1 and Table 2 and Figures S3 and S4) on the identified DEGs were conducted. In the 2.5 μg/L B[a]P treatment, KEGG pathway analysis of the testicular transcriptome highlighted molecular pathways related to (I) male reproduction systems such as GnRH signaling pathway, thyroid hormone signaling pathway, calcium signaling pathway, and apoptosis—multiple species; (II) signaling pathways associated with energy supply and metabolism, such as PPAR signaling pathway, fatty acid metabolism, insulin secretion, glucagon signaling pathway, pantothenate and CoA biosynthesis, propanoate metabolism, butanoate metabolism, starch and sucrose metabolism, peroxisome, and beta-alanine metabolism (Table 1 and Table S3). Genes such as *ZP3* (zona pellucida sperm-binding protein 3), *TRHR* (thyrotropin-releasing hormone receptor), *DIO1* (iodothyronine deiodinase 1), *HSD11B1L* (hydroxysteroid 11-beta dehydrogenase 1 like), and *SEMA7A* (semaphorin 7A) have been identified to be highly related to male reproductive health.

In the 80 μg/L B[a]P treatment, the KEGG pathway related to (I) male reproduction systems such as the GnRH signaling pathway, steroid hormone biosynthesis, estrogen signaling pathway, calcium signaling pathway, and cAMP signaling pathway; (II) signaling pathways associated with energy supply and metabolism, such as the AMPK signaling pathway, insulin signaling pathway, cholesterol metabolism, glycine, serine and threonine metabolism, cysteine and methionine metabolism, and biosynthesis of amino acids; (III) signaling pathways related to cell structure, adhesion, and motility, which include regulation of actin cytoskeleton, focal adhesion, and tight junction; (IV) signaling pathways related to cell fate regulation, oxidative stress, and cell death, including PI3K-Akt signaling pathway, apoptosis, MAPK signaling pathway, cellular senescence, necroptosis, hippo signaling pathway, glutathione metabolism, and oxytocin signaling pathway (Table 2 and Table S4).

Notably, transcriptomic analysis revealed that the expression of epigenetic regulator genes was dysregulated in F0 adult male fish with embryonic B[a]P exposure, of which five genes, including *EZH1* (enhancer of zeste 1 polycomb repressive complex 2 subunit), *HDAC7* (histone deacetylase 7), *KDM2B* (lysine demethylase 2B), *ING3* (inhibitor of growth family member 3), and *UHRF2* (ubiquitin like with PHD and ring finger domains 2), were upregulated. The expression of *PRMT5* (protein arginine methyltransferase 5), *SMARCA4* (SWI/SNF related, matrix associated, actin dependent regulator of chromatin, subfamily A, member 4), *TDRD7* (tudor domain containing 7), and *NPM1* (nucleophosmin 1) was downregulated.

## 4. Discussion

**Figure 5:**
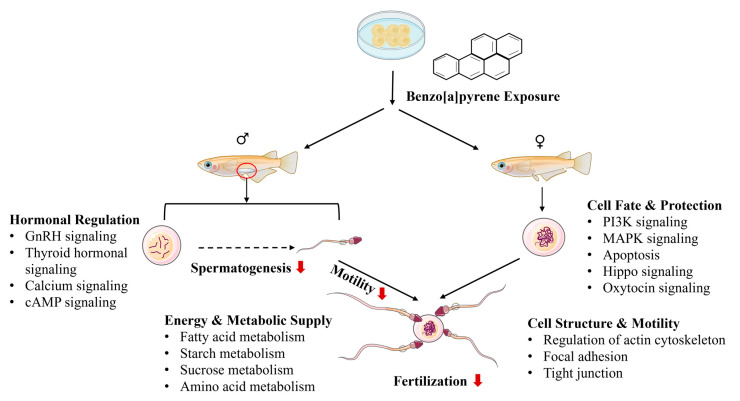
A schematic diagram showing reproductive effects in adult male medaka induced by embryonic benzo[a]pyrene exposure. GSI: gonadosomatic index, HSI: hepatosomatic index. A red arrow indicates suppression of or reduction in certain biological processes.

B[a]P exposure can cause male reproductive toxicity, but the underlying mechanisms remain virtually unknown. In the present study, embryonic exposure to B[a]P induced profound reproductive impairment in adult male medaka, evident by reductions in gonadosomatic index, fertilization capability, and sperms motility. Novel transcriptomic data are presented and linked to the morphometric and physiological changes in B[a]P-induced male reprotoxicity (Figure 5). Four major pathway clusters were associated with reproductive impairment in F0 adult male medaka induced by embryonic B[a]P exposure at environmentally relevant levels: (1) pathways related to hormonal regulation, (2) pathways related to energy metabolism and supply for spermatogenesis, (3) pathways related to sperm cell structure and motility, and (4) pathways related to spermatogenic cell fate and protection. These pathway clusters are potentially regulated by histone modifications.

### 4.1. Hormonal Regulation of Testicular Function

Male reproductive capability is meticulously regulated by the hierarchical brain–pituitary–gonadal axis through stimulatory hormones and negative feedback loops. The pulsatile release of gonadotropin-releasing hormone (GnRH) from the hypothalamus stimulates the anterior pituitary to secrete the gonadotropins luteinizing hormone (LH) and follicle-stimulating hormone (FSH) [38]. LH targets Leydig cells to drive testosterone synthesis and secretion, while FSH acts synergistically with testosterone on Sertoli cells to initiate and maintain spermatogenesis, thereby governing male reproductive function [39]. Testosterone biosynthesis begins with cholesterol mobilization into Leydig cell mitochondria by the StAR protein in response to LH stimulation. A series of enzymatic reactions, primarily via the Δ^5^-pathway involving CYP11A1 and CYP17A1, convert cholesterol to the weak androgen dehydroepiandrosterone, which is subsequently transformed by enzymes including 17β-HSD3 into the potent androgen testosterone [40].

In the present study, embryonic B[a]P exposure induced significant dysregulation of these pathways in adult medaka testes. The 2.5 μg/L treatment enriched pathways including GnRH, thyroid hormone, and calcium signaling. The 80 μg/L treatment dysregulated the GnRH, cyclic AMP (cAMP), and calcium signaling pathways and significantly downregulated key genes encoding steroidogenic enzymes (*CYP17A1*, *HSD3B2*, *CYP1A1*), suggesting suppressed testosterone biosynthesis. This aligns with the observed reductions in gonadosomatic index, sperm motility, and fertilization success and is consistent with the findings that B[a]P exposure reduces plasma testosterone and fertility in other fish models [12].

The calcium and cAMP signaling pathways function as indispensable secondary messengers throughout male reproduction [41,42]. Within the tests, they mediate gonadotropin-stimulated steroidogenesis and spermatogenesis. Furthermore, they are critical for post-testicular sperm function: cAMP regulates sperm capacitation and motility, while calcium is the primary trigger for the acrosome reaction and hyperactivation, with both pathways acting synergistically [42,43]. Consequently, the reproductive impairments observed here are likely substantially attributed to the dysregulation of these crucial signaling pathways in the testes.

### 4.2. Energy and Metabolic Support for Spermatogenesis

Spermatogenesis and sperm motility impose immense bioenergetic demands, met through an integrated network of metabolic pathways that supply ATP and essential biosynthetic precursors. In this study, embryonic B[a]P exposure disrupted these critical processes. Treatments altered key molecular signaling pathways: the 2.5 μg/L exposure enriched pathways for fatty acid, starch, sucrose, and amino acid metabolism, while the 80 μg/L exposure significantly dysregulated central energy and biosynthetic pathways, including AMPK, insulin, and glucagon signaling, cholesterol metabolism, and the metabolism of multiple amino acids.

The AMPK signaling pathway, a central cellular energy sensor, promotes catabolic processes to sustain the high ATP requirements for sperm motility [44]. This is synergistically supported by insulin and glucagon signaling, which govern systemic glucose uptake and utilization. Proper insulin sensitivity is paramount for testicular function, as insulin resistance disrupts the metabolic activity of Sertoli and Leydig cells, providing a mechanistic link between metabolic dysfunction and infertility [45]. Furthermore, cholesterol metabolism fulfills a dual role, serving as the essential precursor for testosterone synthesis and as a vital structural component of sperm membranes, modulating fluidity and stability essential for fertilization [46].

Critically, amino acid metabolism was profoundly affected. Metabolism of glycine, serine, threonine, cysteine, and methionine provides fundamental substrates for protein synthesis and antioxidant defense, and certain amino acids act as nitric oxide precursors to regulate sperm function [47,48]. The predominant downregulation of key genes (e.g., *GAMT*, *BHMT*, *CBS*, *AHCY*, *MAT1A*, *ALDH3B1*) in the 80 μg/L treatment indicates a severe impairment of these metabolic functions. The coordinated dysregulation of these essential pathways likely failed to create the tailored metabolic environment required to support the biosynthetic and energetic challenges of spermatogenesis, contributing to the observed reproductive impairment in adult male medaka. The alterations in energy metabolism and supply for testicular tissue of adult fish at the metabolic level are to be validated with metabolomic analysis in subsequent studies.

### 4.3. Sperm Cell Structure and Motility

The structural and functional transformations essential for spermatogenesis, particularly the remodeling of a spermatid into a motile spermatozoon during spermiogenesis, are precisely executed by pathways governing the cytoskeleton, adhesion, and motility [49,50,51]. In the present study, embryonic B[a]P exposure at 80 μg/L dysregulated key pathways including the regulation of actin cytoskeleton, focal adhesion, and tight junctions.

The actin cytoskeleton provides the primary mechanical force for cellular metamorphosis, driving nuclear elongation, acrosome formation, and cytoplasmic expulsion [52]. This cytoskeletal dynamism is equally critical for Sertoli cell function, enabling the constant remodeling of the blood–testis barrier and the translocation of developing germ cells [51]. This barrier is composed of tight junctions that form impenetrable seals between Sertoli cells to create an immune-privileged microenvironment that protects meiotic and post-meiotic germ cells and maintains a specialized fluid milieu essential for successful spermatogenesis [49,53]. Simultaneously, focal adhesion pathways mediate the robust attachments between Sertoli cells and the basement membrane and between Sertoli cells and germ cells. These adhesions act as vital signaling hubs that coordinate cell survival, polarity, and migration [50]. The integrated function of these systems—where the actin cytoskeleton provides motive force, focal adhesions provide attachment and communication, and tight junctions provide a protected space—ensures the structural integrity of the seminiferous epithelium and the production of functional sperm [49,50,51]. Interestingly, the genes enriched in these pathways were exclusively upregulated, which may represent a compensatory response to the impairment of spermatogenesis and sperm motility. Nonetheless, further histopathological analysis is warranted to validate these molecular findings.

### 4.4. Spermatogenic Cell Fate and Protection

Spermatogenesis requires a precise balance between cell survival, proliferation, and programmed death to ensure sufficient production of high-quality sperm. In this study, embryonic B[a]P exposure dysregulated key signaling pathways governing cell fate, including PI3K-Akt, apoptosis, MAPK, Hippo, and oxytocin signaling, in the 80 μg/L treatment. Apoptosis pathways were also enriched in the 2.5 μg/L group.

The PI3K-Akt pathway acts as a central guardian of cell fate, promoting survival and proliferation while inhibiting apoptosis in both germ and somatic cells [54]. This survival signal is counterbalanced by apoptosis, which serves as an essential quality control mechanism by eliminating damaged or abnormal germ cells [55]. Here, B[a]P exposure activated apoptotic pathways, which correlates with observed reductions in testis weight, sperm motility, and fertilization capability. These findings align with studies in tilapia and rodent models where B[a]P exposure induced testicular apoptosis and reproductive impairment [21,23,31,56,57,58,59,60]. B[a]P-induced oxidative stress is not only linked to DNA damage and apoptotic cell death but also associated with disruptions of proteins that organize and protect DNA, including histones and their chemical modifications, exerting profound multigenerational effects [1]. Interestingly, our data show that several genes encoding histone modification enzymes were dysregulated in F0 adult male fish with embryonic B[a]P, and what regulatory roles histone modifications play warrant further in-depth studies.

The MAPK signaling pathway integrates extracellular cues, including those from FSH and testosterone, to regulate proliferation, differentiation, and stress adaptation [57,61]. Meanwhile, the Hippo pathway regulates testis size and structural integrity by controlling Sertoli cell proliferation and blood–testis barrier dynamics [62,63]. Finally, oxytocin signaling potentiates testosterone synthesis and may facilitate sperm delivery through reproductive tract contractions [64]. Collectively, the dysregulation of these critical pathways likely disrupted the delicate balance between cell survival and death, compromising both sperm quantity and quality control mechanisms, as evidenced by the reduced sperm motility and fertilization success observed in this study.

### 4.5. Limitations of This Study

Regarding the reproductive toxicity observed in adult male fish stemming from embryonic B[a]P exposure, the bioavailability of B[a]P to fish embryos is a critical consideration. Fish embryos can absorb B[a]P directly from the surrounding water; therefore, absorption efficiency, internal metabolism, and potential excretion or degradation of B[a]P within the embryo may substantially influence its eventual toxicity. Several studies have attempted to quantify B[a]P and its metabolites in fish embryos following direct or parental exposure [9,11,33,65]. To better understand the bioavailability and toxicity of B[a]P, future work should aim to determine the internal concentrations of B[a]P in embryos using GC-MS, as described by Mo et al. (2020) [11]. With respect to the effects of embryonic B[a]P exposure on adult sperm parameters, while sperm motility data are presented, sperm morphology and concentration remain unassessed. A histological examination of the testes would offer valuable insights into potential structural alterations. Furthermore, functional validation of the molecular findings is essential to establish a causal link between embryonic B[a]P exposure and impaired reproductive function in adult males. These key aspects of male reproductive capacity represent important directions for future research.

While the reported recovery of reproductive capacity in the F1 generation is an important finding, the discussion lacks mechanistic depth. Whether this recovery represents a true molecular reset or merely a physiological compensation are not explored. The absence of transcriptomic or epigenetic data from F1 testes is a significant limitation, as it could reveal compensatory pathways or residual subtler dysregulations. Crucially, the assessment of recovery was limited to gross reproductive parameters; it did not evaluate more latent threats such as sperm DNA damage or mutational load in the F1 germline, which could manifest as defects in the F2 generation. Therefore, investigations of these underlying mechanisms and longer-term generational health are required.

## 5. Conclusions

Embryonic exposure to environmentally relevant levels of B[a]P impaired the reproductive fitness of F0 adult medaka but not F1 adult fish. B[a]P exposure reduced the percentage of motile sperms and sperm motility, which were closely associated with transcriptional dysregulation in the testicular tissues of medaka adults. These dysregulated molecular signaling pathways were related to hormonal metabolism and regulation, energy and metabolic support for spermatogenesis, sperm cell structure and motility, and spermatogenic cell fate and protection. Interestingly, multiple genes that encode histone modification enzymes were differentially expressed, suggesting the potential involvement of histone modifications in B[a]P-induced reprotoxicity in male fish. These results suggest that the health risk of B[a]P imposed on fish warrants more attention. Transcriptomic analysis serves as a powerful complement to conventional phenotypic endpoint measurements. Conserved molecular pathways may also be present and vital in humans, and these findings act as a powerful hypothesis generator for human health and remain to be validated.

## Figures and Tables

**Table 1:** Significantly altered KEGG pathways in testes of matured medaka with embryonic benzo[a]pyrene exposure at 2.5 μg/L for 8 days.

Pathways	ID	*p*-Value	Up-DEGs	Down-DEGs
PPAR signaling pathway	hsa03320	0.000152	*ACSL5*	*ACOX3*, *CYP8B1*
Drug metabolism—other enzymes	hsa00983	0.00017	-	*UPB1*, *UGT2B17*, *XDH*
Fatty acid metabolism	hsa01212	0.002695	*ACSL5*	*ACOX3*
Insulin secretion	hsa04911	0.005887	-	*PLCB1*, *ABCC8*
GnRH signaling pathway	hsa04912	0.006827	-	*PLCB1*, *MAP2K6*
Glucagon signaling pathway	hsa04922	0.008741	-	*PLCB1*, *G6PC1*
Thyroid hormone signaling pathway	hsa04919	0.010865	-	*PLCB1*, *DIO1*
Primary bile acid biosynthesis	hsa00120	0.023111	-	*CYP8B1*
Calcium signaling pathway	hsa04020	0.026641	*TRHR*	*PLCB1*
Apoptosis—multiple species	hsa04215	0.043213	-	*DIABLO*

**Table 2:** Significantly altered KEGG pathways in testes of matured medaka with embryonic benzo[a]pyrene exposure at 80 μg/L for 8 days.

Pathways	ID	*p*-Value	Up-DEGs	Down-DEGs
Glycine, serine and threonine metabolism	hsa00260	1.63 × 10^−5^	*PGAM2*	*GAMT*, *BHMT*, *PIPOX*, *CBS*, *DMGDH*
Tight junction	hsa04530	0.000295	*CLDN11*, *PARD6G*, *ARHGEF2*, *CACNA1D*, *EZR*, *MSN*, *TJP1*, *VASP*, *CFTR*	**-**
PI3K-Akt signaling pathway	hsa04151	0.0004735	*PDGFA*, *PDGFRA*, *ITGB8*, *LAMC1*, *ITGA7*	*COL6A2*, *G6PC1*, *THBS1*, *MAP2K2*, *NGFR*, *ATF4*, *MYC*, *ANGPT4*
Focal adhesion	hsa04510	0.000871	*PDGFA*, *PDGFRA*, *ITGB8*, *ITGA7*, *PAK5*, *VASP*, *LAMC1*	*COL6A2*, *THBS1*
GnRH signaling pathway	hsa04912	0.0011547	*ADCY5, CACNA1D, PTK2B, MAPK12*	*MAP2K2*, *ATF4*
Regulation of actin cytoskeleton	hsa04810	0.0014148	*PDGFA*, *PDGFRA*, *ITGB8*, *EZR*, *ITGA7*, *MSN*, *ARPC1B*, *PAK5*	*MAP2K2*
Cysteine and methionine metabolism	hsa00270	0.0035349	**-**	*CBS*, *AHCY*, *MAT1A*, *BHMT*
AMPK signaling pathway	hsa04152	0.0038957	*LIPE*, *PFKFB1*, *TBC1D1*, *PFKFB4*, *CFTR*	*G6PC1*
Gap junction	hsa04540	0.0049489	*PDGFA*, *PDGFRA*, *TJP1*, *ADCY5*	*MAP2K2*
Steroid hormone biosynthesis	hsa00140	0.0069325	*AKR1D1*	*CYP1A1*, *HSD3B2*, *CYP17A1*
beta-Alanine metabolism	hsa00410	0.0087031	**-**	*ALDH3B2*, *ALDH6A1*, *ABAT*
Oxytocin signaling pathway	hsa04921	0.0117059	*ADCY5*, *CACNA1D*, *CACNG4*	*CD38*, *MAP2K2*
Cellular senescence	hsa04218	0.0142243	*CACNA1D*, *SMAD2*, *MAPK12*	*MAP2K2*, *MYC*
cAMP signaling pathway	hsa04024	0.0163771	*ADCY5*, *PPP1R1B*, *LIPE*, *CACNA1D*, *RAPGEF4*, *CFTR*	*MAP2K2*
Cholesterol metabolism	hsa04979	0.0246684	*ABCA1*	*APOB*, *APOA1*
Apoptosis	hsa04210	0.0261614	*CTSF*	*MAP2K2*, *PARP1*, *DIABLO*, *ATF4*
Insulin signaling pathway	hsa04910	0.0268635	*LIPE*, *PRKAR1B*	*MAP2K2*, *G6PC1*, *SOCS3*
Estrogen signaling pathway	hsa04915	0.0275775	*MMP9*, *PGR*, *ADCY5*	*MAP2K2*, *ATF4*
Calcium signaling pathway	hsa04020	0.03121	*PDGFRA*, *TPCN1*, *CACNA1D*, *PLCG1*, *PTK2B*	*CD38*
Glutathione metabolism	hsa00480	0.0325229	**-**	*CHAC1*, *ANPEP*, *RRM2*
Cell adhesion molecules	hsa04514	0.0337238	*CLDN11*, *ITGB8*, *F3*, *CADM3*	*CD34*
Endocrine resistance	hsa01522	0.0328224	*MMP9*, *MAPK12*, *ADCY5*	*MAP2K2*
Hippo signaling pathway	hsa04390	0.0406579	*PARD6G*, *FZD3*, *SMAD2*	*MYC*, *GDF7*
Glucagon signaling pathway	hsa04922	0.0414946	*PFKFB1*, *PGAM2*	*ATF4*, *G6PC1*
Necroptosis	hsa04217	0.0483953	*STAT6*, *GLUL*, *SPATA2L*	*PPIA*, *PARP1*

## Data Availability

Transcriptomic raw data were deposited in NCBI database with an assigned BioProject accession number PRJNA1328451. [NCBI] [https://www.ncbi.nlm.nih.gov/] [PRJNA1328451].

## Supplementary Materials

The following supporting information can be downloaded at: https://www.mdpi.com/article/10.3390/toxics13100886/s1, Figure S1: Multigenerational effects of embryonic benzo[a]pyrene exposure on adult female morphometry. Fulton’s condition factor (a), hepatosomatic index (b), and gonadosomatic index (c) of F0 and F1 adult medaka were impacted by an 8-day embryonic exposure to benzo[a]pyrene. Values represent mean ± SEM (n = 4), and statistical analysis was performed using a two-way ANOVA with Tukey’s post hoc test.; Figure S2: Altered fecundity in F0 and F1 adult female medaka after embryonic benzo[a]pyrene exposure. Embryos were exposed to benzo[a]pyrene (Con, 2.5, 20, or 80 μg/L) for 8 days. Upon reaching adulthood, the reproductive performance of the directly exposed generation (F0) and their offspring (F1) was evaluated. Data are shown as mean ± SEM (n = 4), and statistical analysis was performed using a two-way ANOVA with Tukey’s post hoc test.; Figure S3: Gene Ontology (GO) enrichment analysis of F0 adult male medaka testes with embryonic benzo[a]pyrene exposure at 2.5 μg/L using the Database for Annotation, Visualization and Integrated Discovery (DAVID). (a) Enriched biological processes (BP), (b) enriched cellular components (CC), and (c) enriched molecular functions (MFs) are shown.; Figure S4: Gene Ontology (GO) enrichment analysis of F0 adult male medaka testes with embryonic benzo[a]pyrene exposure at 80 μg/L using the Database for Annotation, Visualization and Integrated Discovery (DAVID). (a) Enriched biological processes (BP), (b) enriched cellular components (CC), and (c) enriched molecular functions (MF) are shown.; Table S1: A two-way ANOVA was performed to compare the fertilization capability of F0 and F1 adult medaka following embryonic benzo[a]pyrene exposure.; Table S2: Transcriptomic raw data of F0 adult male medaka testicular tissues with and without embryonic benzo[a]pyrene exposure.; Table S3: Significantly altered KEGG pathways in testes of matured medaka with embryonic benzo[a]pyrene exposure at 2.5 μg/L for 8 days.; Table S4: Significantly altered KEGG pathways in testes of matured medaka with embryonic benzo[a]pyrene exposure at 80 μg/L for 8 days.

## References

[1] Mo J., Au D.W.T., Guo J., Winkler C., Kong R.Y.C., Seemann F. (2022). Benzo[a]pyrene osteotoxicity and the regulatory roles of genetic and epigenetic factors: A review. Crit. Rev. Environ. Sci. Technol..

[2] Singare P.U. (2016). Carcinogenic and endocrine-disrupting PAHs in the aquatic ecosystem of India. Environ. Monit. Assess..

[3] Ekere N.R., Yakubu N.M., Oparanozie T., Ihedioha J.N. (2019). Levels and risk assessment of polycyclic aromatic hydrocarbons in water and fish of Rivers Niger and Benue confluence Lokoja, Nigeria. J. Environ. Health Sci. Eng..

[4] Mojiri A., Zhou J.L., Ohashi A., Ozaki N., Kindaichi T. (2019). Comprehensive review of polycyclic aromatic hydrocarbons in water sources, their effects and treatments. Sci. Total Environ..

[5] Bukowska B., Mokra K., Michałowicz J. (2022). Benzo[a]pyrene—Environmental occurrence, human exposure, and mechanisms of toxicity. Int. J. Mol. Sci..

[6] Yang S.K., Deutsch J.O.S.E.P.H., Gelboin H.V. (2012). Benzo[a]pyrene metabolism: Activation and detoxification. Polycycl. Hydrocarb. Cancer.

[7] Chepelev N.L., Moffat I.D., Bowers W.J., Yauk C.L. (2015). Neurotoxicity may be an overlooked consequence of benzo[a]pyrene exposure that is relevant to human health risk assessment. Mutat. Res./Rev. Mutat. Res..

[8] Mo J., Chen Y., Lai K.P., Seemann F., Liu W. (2025). Benzo[a]pyrene osteotoxicity, neurotoxicity, and epigenetic effects in fishes and mammals: A review. Environ. Chem. Lett..

[9] Corrales J., Thornton C., White M., Willett K.L. (2014). Multigenerational effects of benzo[a]pyrene exposure on survival and developmental deformities in zebrafish larvae. Aquat. Toxicol..

[10] Caiment F., Gaj S., Claessen S., Kleinjans J. (2015). High-throughput data integration of RNA–miRNA–circRNA reveals novel insights into mechanisms of benzo[a]pyrene-induced carcinogenicity. Nucleic Acids Res..

[11] Mo J., Au D.W.-T., Wan M.T., Shi J., Zhang G., Winkler C., Kong R.Y.-C., Seemann F. (2020). Multigenerational impacts of benzo[a]pyrene on bone modeling and remodeling in medaka (*Oryzias latipes*). Environ. Sci. Technol..

[12] Booc F., Thornton C., Lister A., MacLatchy D., Willett K.L. (2014). Benzo[a]pyrene effects on reproductive endpoints in *Fundulus heteroclitus*. Toxicol. Sci..

[13] Yang Y., Pan L., Zhou Y., Xu R., Li D. (2020). Benzo[a]pyrene exposure disrupts steroidogenesis and impairs spermatogenesis in diverse reproductive stages of male scallop (*Chlamys farreri*). Environ. Res..

[14] Ramesh A., Harris K.J., Archibong A.E. (2022). Reproductive toxicity of polycyclic aromatic hydrocarbons. Reproductive and Developmental Toxicology.

[15] Montano L., Baldini G.M., Piscopo M., Liguori G., Lombardi R., Ricciardi M., Esposito G., Pinto G., Fontanarosa C., Spinelli M. (2025). Polycyclic aromatic hydrocarbons (PAHs) in the environment: Occupational exposure, health risks and fertility implications. Toxics.

[16] Bouayed J., Desor F., Soulimani R. (2009). Subacute oral exposure to benzo[a]pyrene (B[a]P) increases aggressiveness and affects consummatory aspects of sexual behaviour in male mice. J. Hazard. Mater..

[17] Mohamed E.-S.A., Song W.-H., Oh S.-A., Park Y.-J., You Y.-A., Lee S., Choi J.-Y., Kim Y.-J., Jo I., Pang M.-G. (2010). The transgenerational impact of benzo[a]pyrene on murine male fertility. Hum. Reprod..

[18] Chung J.Y., Kim Y.J., Kim J.Y., Lee S.G., Park J.E., Kim W.R., Kim J.M. (2011). Benzo[a]pyrene reduces testosterone production in rat Leydig cells via a direct disturbance of testicular steroidogenic machinery. Environ. Health Perspect..

[19] Liang J., Zhu H., Li C., Ding Y., Zhou Z., Wu Q. (2012). Neonatal exposure to benzo[a]pyrene decreases the levels of serum testosterone and histone H3K14 acetylation of the StAR promoter in the testes of SD rats. Toxicology.

[20] Xu G., McMahan C.A., Walter C.A. (2014). Early-life exposure to benzo[a]pyrene increases mutant frequency in spermatogenic cells in adulthood. PLoS ONE.

[21] Ling X., Yang W., Zou P., Zhang G., Wang Z., Zhang X., Ao L. (2018). TERT regulates telomere-related senescence and apoptosis through DNA damage response in male germ cells exposed to BPDE in vitro and to B[a]P in vivo. Environ. Pollut..

[22] Godschalk R.W., Verhofstad N., Verheijen M., Yauk C.L., Linschooten J.O., van Steeg H., van Schooten F.J. (2015). Effects of benzo[a]pyrene on mouse germ cells: Heritable DNA mutation, testicular cell hypomethylation and their interaction with nucleotide excision repair. Toxicol. Res..

[23] Jeng H.A., Yordt D., Davis S., Swanson J.R. (2015). Assessment of alteration of reproductive system in vivo induced by subchronic exposure to benzo[a]pyrene via oral administration. Environ. Toxicol..

[24] Jorge B.C., Reis A.C.C., Stein J., da Silva Balin P., Sterde E.T., Barbosa M.G., Arena A.C. (2021). Parental exposure to benzo[a]pyrene in the peripubertal period impacts reproductive aspects of the F1 generation in rats. Reprod. Toxicol..

[25] Jorge B.C., Reis A.C.C., Sterde É.T., da Silva Balin P., Scarano W.R., Hisano H., Arena A.C. (2021). Exposure to benzo[a]pyrene from juvenile period to peripubertal impairs male reproductive parameters in adult rats. Chemosphere.

[26] O’Brien J.M., Beal M.A., Yauk C.L., Marchetti F. (2016). Benzo[a]pyrene is mutagenic in mouse spermatogonial stem cells and dividing spermatogonia. Toxicol. Sci..

[27] Zhang W., Yang J., Lv Y., Li S., Qiang M. (2019). Paternal benzo[a]pyrene exposure alters the sperm DNA methylation levels of imprinting genes in F0 generation mice and their unexposed F1-2 male offspring. Chemosphere.

[28] Zhang C., Ma Y., Liu W., Ma S., Chen Z., Hao X., Wang Z. (2024). ranscriptomic and proteomic features of a mouse model of sperm DNA damage induced by benzo[a]pyrene. Reprod. Toxicol..

[29] Zhang L., Chen W.Q., Han X.Y., Wang H.L., Gao P.Z., Wang D.M., Liu S.Z. (2024). Benzo[a]pyrene exposure during pregnancy leads to germ cell apoptosis in male mice offspring via affecting histone modifications and oxidative stress levels. Sci. Total Environ..

[30] Kennedy C.J., Smyth K.R. (2015). Disruption of the rainbow trout reproductive endocrine axis by the polycyclic aromatic hydrocarbon benzo[a]pyrene. Gen. Aomparative Andocrinology.

[31] Colli-Dula R.C., Fang X., Moraga-Amador D., Albornoz-Abud N., Zamora-Bustillos R., Conesa A., Hernandez-Nuñez E. (2018). Transcriptome analysis reveals novel insights into the response of low-dose benzo[a]pyrene exposure in male tilapia. Aquat. Toxicol..

[32] Xu K., Gao D., Lin J., Dai Q., Zhou Q., Chen Y., Wang C. (2023). Benzo[a]pyrene exposure in early life suppresses spermatogenesis in adult male zebrafish and association with the methylation of germ cell-specific genes. Aquat. Toxicol..

[33] Zeb R., Yin X., Chen F., Bo J., Wang K.J. (2025). Life-cycle benzo[a]pyrene exposure induces sex-specific reproductive impairment, feminization, and transgenerational disruption in marine medaka (*Oryzias melastigma*). Environ. Sci. Technol..

[34] Mo J., Wan M.T., Au D.W.T., Shi J., Tam N., Qin X., Seemann F. (2023). Transgenerational bone toxicity in F3 medaka (*Oryzias latipes*) induced by ancestral benzo[a]pyrene exposure: Cellular and transcriptomic insights. J. Environ. Sci..

[35] Kinoshita M., Murata K., Naruse K., Tanaka M. (2009). Medaka: Biology, Management, and Experimental Protocols.

[36] Organisation for Economic Co-operation and Development (1992). OECD Guideline for Testing of Chemicals.

[37] Qin X., Lai K.P., Wu R.S.S., Kong R.Y.C. (2022). Continuous 17α-ethinylestradiol exposure impairs the sperm quality of marine medaka (*Oryzias melastigma*). Mar. Pollut. Bull..

[38] Plant T.M. (2015). 60 Years of neuroendocrinology: The hypothalamo-pituitary–gonadal axis. J. Endocrinol..

[39] Acevedo-Rodriguez A., Kauffman A.S., Cherrington B.D., Borges C.S., Roepke T.A., Laconi M. (2018). Emerging insights into hypothalamic-pituitary-gonadal axis regulation and interaction with stress signalling. J. Neuroendocrinol..

[40] Hu J., Zhang Z., Shen W.-J., Azhar S. (2010). Cellular cholesterol delivery, intracellular processing and utilization for biosynthesis of steroid hormones. Nutr. Metab..

[41] Wang H., McGoldrick L.L., Chung J.-J. (2021). Sperm ion channels and transporters in male fertility and infertility. Nat. Rev. Urol..

[42] Rahman M.S., Kwon W.S., Pang M.G. (2014). Calcium influx and male fertility in the context of the sperm proteome: An update. BioMed Res. Int..

[43] Stewart T.A., Davis F.M. (2019). An element for development: Calcium signaling in mammalian reproduction and development. Biochim. Biophys. Acta (BBA)-Mol. Cell Res..

[44] Yang W., Wang L., Wang F., Yuan S. (2020). Roles of AMP-activated protein kinase (AMPK) in mammalian reproduction. Front. Cell Dev. Biol..

[45] Cannarella R., Condorelli R.A., La Vignera S., Calogero A.E. (2018). Effects of the insulin-like growth factor system on testicular differentiation and function: A review of the literature. Andrology.

[46] Shi J.F., Li Y.K., Ren K., Xie Y.J., Yin W.D., Mo Z.C. (2018). Characterization of cholesterol metabolism in Sertoli cells and spermatogenesis. Mol. Med. Rep..

[47] Dai Z., Wu Z., Hang S., Zhu W., Wu G. (2015). Amino acid metabolism in intestinal bacteria and its potential implications for mammalian reproduction. MHR Basic Sci. Reprod. Med..

[48] Cui Y., Han J., Ren J., Chen H., Xu B., Song N., Shen G. (2019). Untargeted LC-MS-based metabonomics revealed that aristolochic acid I induces testicular toxicity by inhibiting amino acids metabolism, glucose metabolism, β-oxidation of fatty acids and the TCA cycle in male mice. Toxicol. Appl. Pharmacol..

[49] Mital P., Hinton B.T., Dufour J.M. (2011). The blood-testis and blood-epididymis barriers are more than just their tight junctions. Biol. Reprod..

[50] Roa-Espitia A.L., Hernández-Rendón E.R., Baltiérrez-Hoyos R., Muñoz-Gotera R.J., Cote-Vélez A., Jiménez I., Hernández-González E.O. (2016). Focal adhesion kinase is required for actin polymerization and remodeling of the cytoskeleton during sperm capacitation. Biol. Open.

[51] Breitbart H., Finkelstein M. (2018). Actin cytoskeleton and sperm function. Biochem. Biophys. Res. Commun..

[52] Soda T., Miyagawa Y., Fukuhara S., Tanaka H. (2020). Physiological role of actin regulation in male fertility: Insight into actin capping proteins in spermatogenic cells. Reprod. Med. Biol..

[53] Cyr D.G., Dufresne J., Gregory M. (2018). Cellular junctions in the epididymis, a critical parameter for understanding male reproductive toxicology. Reprod. Toxicol..

[54] Deng C.Y., Lv M., Luo B.H., Zhao S.Z., Mo Z.C., Xie Y.J. (2021). The role of the PI3K/AKT/mTOR signalling pathway in male reproduction. Curr. Mol. Med..

[55] Xu Z.J., Liu M., Niu Q.J., Huang Y.X., Zhao L., Lei X.G., Sun L.H. (2023). Both selenium deficiency and excess impair male reproductive system via inducing oxidative stress-activated PI3K/AKT-mediated apoptosis and cell proliferation signaling in testis of mice. Free Radic. Biol. Med..

[56] Banerjee B., Chakraborty S., Ghosh D., Raha S., Sen P.C., Jana K. (2016). Benzo[a]pyrene induced p53 mediated male germ cell apoptosis: Synergistic protective effects of curcumin and resveratrol. Front. Pharmacol..

[57] Banerjee B., Nandi P., Chakraborty S., Raha S., Sen P.C., Jana K. (2016). Resveratrol ameliorates benzo[a]pyrene-induced testicular dysfunction and apoptosis: Involvement of p38 MAPK/ATF2/iNOS signaling. J. Nutr. Biochem..

[58] Yang W., Cui H., Chai Z., Zou P., Shi F., Yang B., Ao L. (2022). Benzo[a]pyrene inhibits testosterone biosynthesis via NDUFA10-mediated mitochondrial compromise in mouse Leydig cells: Integrating experimental and in silico toxicological approaches. Ecotoxicol. Environ. Saf..

[59] Traini G., Tamburrino L., Ragosta M.E., Guarnieri G., Morelli A., Vignozzi L., Baldi E., Marchiani S. (2023). Effects of benzo[a]pyrene on human sperm functions: An in vitro study. Int. J. Mol. Sci..

[60] Zhang L., Ji X., Ding F., Wu X., Tang N., Wu Q. (2022). Apoptosis and blood-testis barrier disruption during male reproductive dysfunction induced by PAHs of different molecular weights. Environ. Pollut..

[61] Luo D., He Z., Yu C., Guan Q. (2022). Role of p38 MAPK signalling in testis development and male fertility. Oxidative Med. Cell. Longev..

[62] Sharma S.S., Vats A., Majumdar S. (2019). Regulation of Hippo pathway components by FSH in testis. Reprod. Biol..

[63] Kruger R.E., Aziz F., Ralston A. (2025). Hippo signaling in mammalian reproduction. Reproduction.

[64] Stadler B., Whittaker M.R., Exintaris B., Middendorff R. (2020). Oxytocin in the male reproductive tract; the therapeutic potential of oxytocin-agonists and-antagonists. Front. Endocrinol..

[65] Hornung M.W., Cook P.M., Fitzsimmons P.N., Kuehl D.W., Nichols J.W. (2007). Tissue distribution and metabolism of benzo[a]pyrene in embryonic and larval medaka (*Oryzias latipes*). Toxicol. Sci..

