# Supplementary material for: Embryonic Benzo[a]pyrene Exposure Induces Multigenerational Reproductive Effects on Adult Male Medaka: Phenotypic and Transcriptomic Insights
Source: Toxics. 2025 Oct 17;13(10):886. doi: 10.3390/toxics13100886 (PMC12567718; doi:10.3390/toxics13100886)
Supplement: Supplementary file 1 [file toxics-13-00886-s001.zip › toxics-3905713-supplementary.pdf]

Supplementary Materials

Embryonic Benzo[a]pyrene Exposure Induces Multigenerational Reproductive Effects on Adult Male Medaka: Phenotypic and Transcriptomic Insights

Yinhua Chen[1,†], Yi Yang[2,†], Xian Qin[3], Jiangang Wang[1], Guanglong Tang[1], Rim EL Amouri[1], Jiayang Chen[4], Jack Chi-Ho Ip[5], Wenhua Liu[1,6], Jiezhang Mo[1,6]*

1 Guangdong Provincial Key Laboratory of Marine Disaster Prediction and Prevention, Shantou University, Shantou, 515063, China

2 Department of Infectious Diseases and Public Health, City University of Hong Kong, Hong Kong SAR, China

3 State Key Laboratory of Marine Environmental Health, City University of Hong Kong, Hong Kong SAR, China

4 Central Laboratory, Shantou University, Shantou 515063, China

5 Science Unite, Lingnan University, Hong Kong SAR, China

6 International Joint Research Center for Marine Ecological Protection and Disaster Prevention, Shantou University, Shantou 515063, China

† These authors contributed equally to this work

* Corresponding Author:

Jiezhang Mo (PhD)

Address for Communication: Department of Biology, College of Science, Shantou University, Guangdong, China. 515063, Phone: +86-18320390437. E-mail: jzhmo@stu.edu.cn

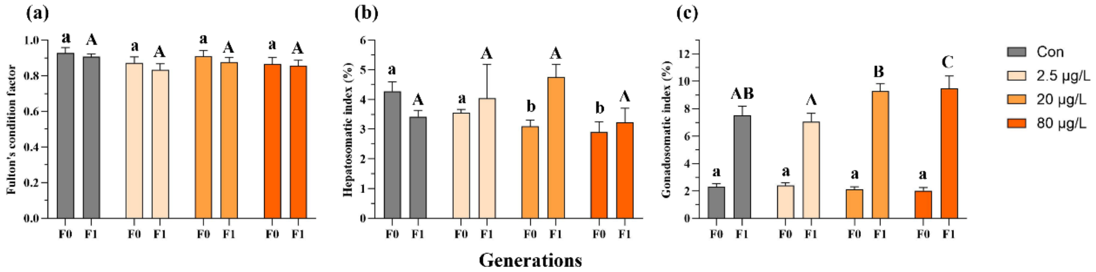

Figure S1. Multigenerational effects of embryonic benzo[a]pyrene exposure on adult female morphometry. The Fulton's condition factor (a), hepatosomatic index (b), and gonadosomatic index (c) of F0 and F1 adult medaka were impacted by an 8-day embryonic exposure to benzo[a]pyrene. Values represent mean ± SEM (n=4), and statistical analysis was performed using a two-way ANOVA with Tukey's post hoc test.

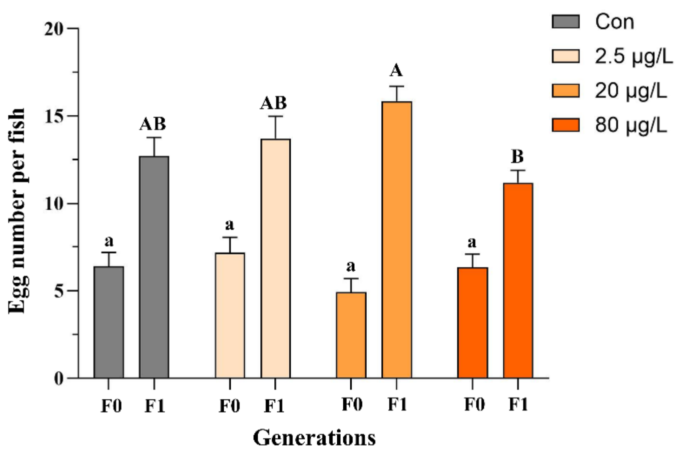

Figure S2. Altered fecundity in F0 and F1 adult female medaka after embryonic benzo[a]pyrene exposure. Embryos were exposed to benzo[a]pyrene (Con, 2.5, 20, or 80 μg/L) for 8 days. Upon reaching adulthood, the reproductive performance of the directly exposed generation (F0) and their offspring (F1) was evaluated. Data are shown as mean ± SEM (n = 4), and statistical analysis was performed using a two-way ANOVA with Tukey's post hoc test.

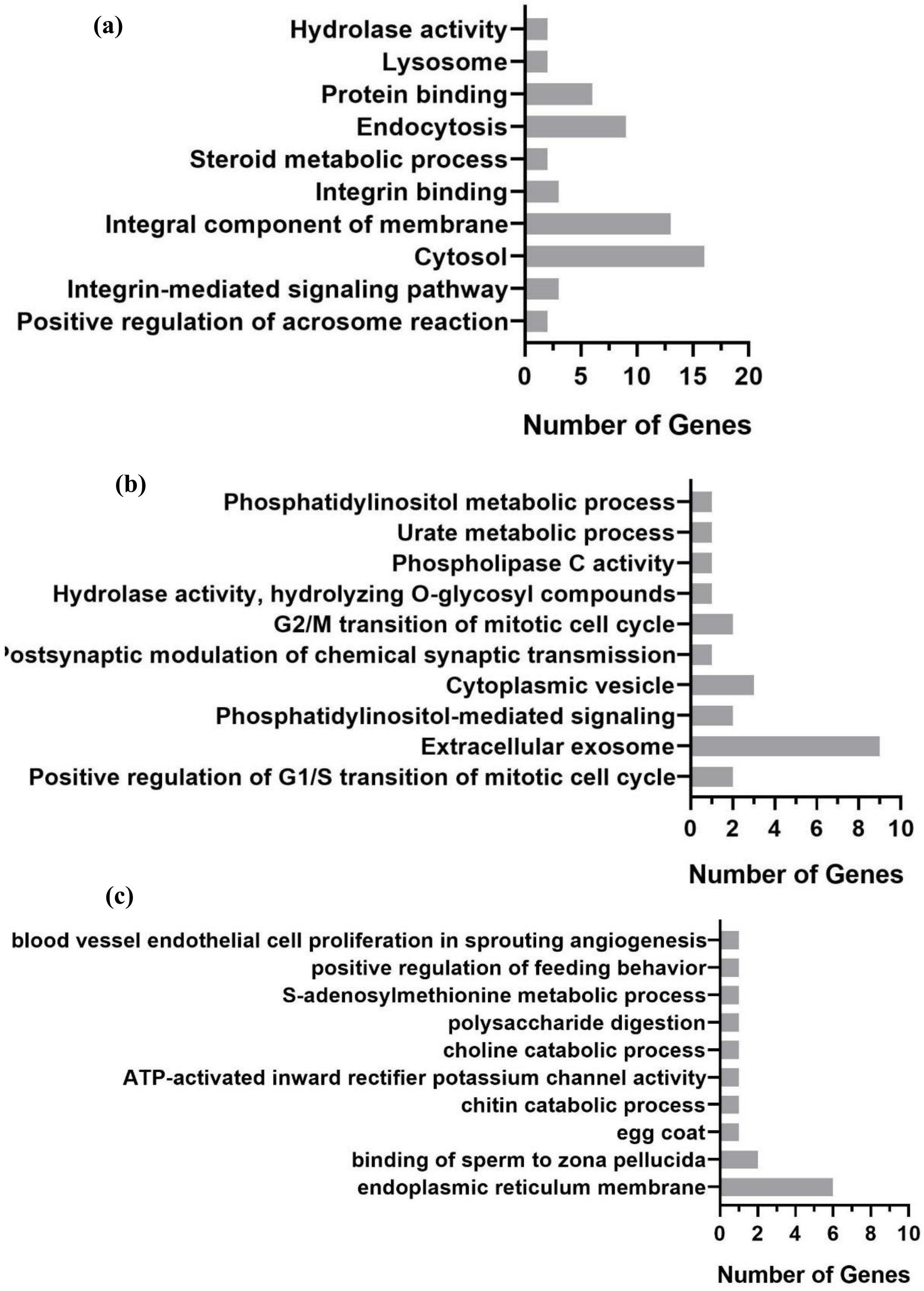

Figure S3. Gene Ontology (GO) enrichment analysis of F0 adult male medaka testes with embryonic benzo[a]pyrene exposure at 2.5 μg/L using the Database for Annotation, Visualization and Integrated Discovery (DAVID). (a) Enriched biological processes (BP), (b) enriched cellular components (CC), and (c) enriched molecular functions (MF) are shown.

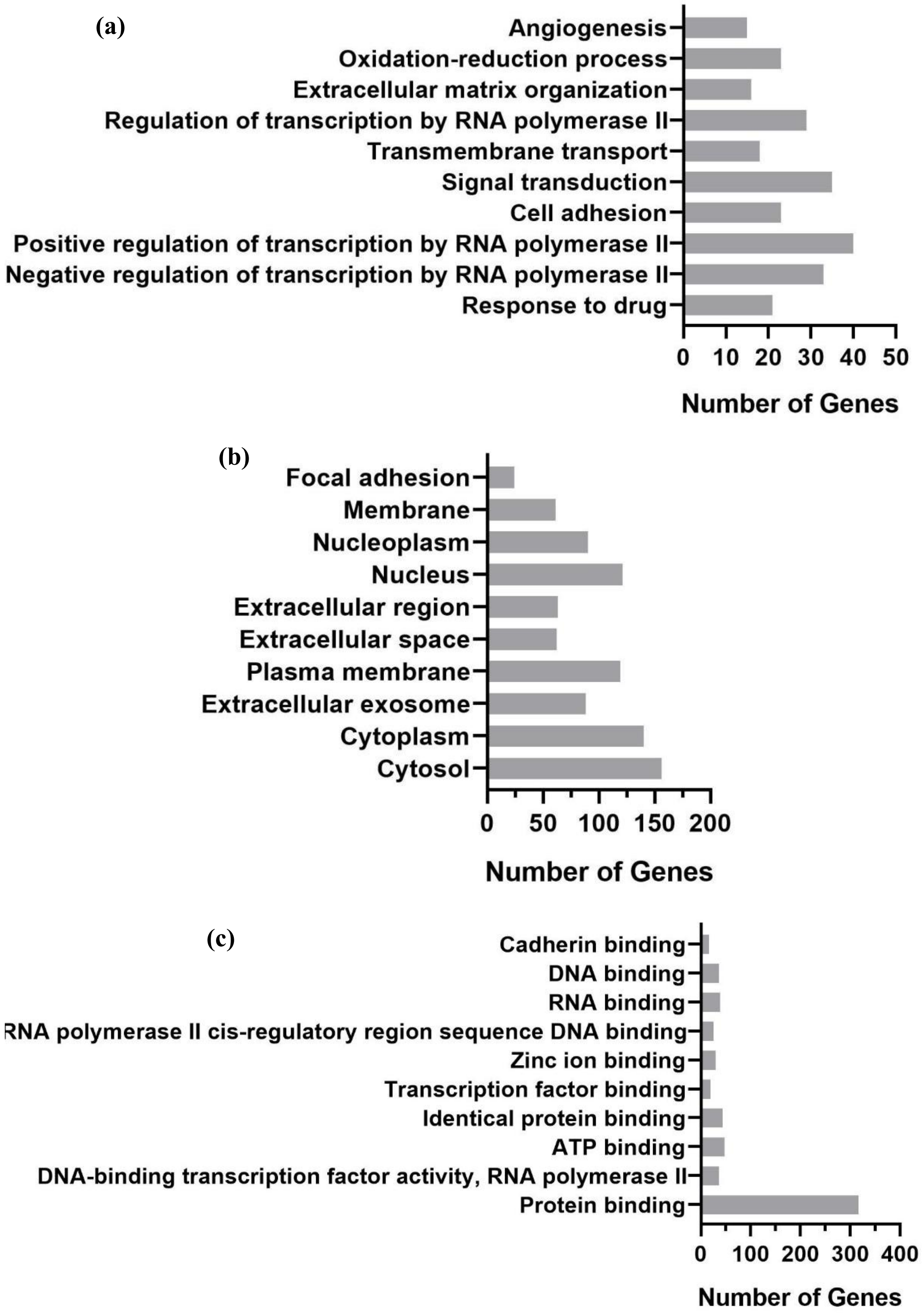

Figure S4. Gene Ontology (GO) enrichment analysis of F0 adult male medaka testes with embryonic benzo[a]pyrene exposure at 80 μg/L using the Database for Annotation, Visualization and Integrated Discovery (DAVID). (a) Enriched biological processes (BP), (b) enriched cellular components (CC), and (c) enriched molecular functions (MF) are shown.

Table S1. A two-way ANOVA was performed to compare the fertilization capability of F0 and F1 adult medaka following embryonic benzo[a]pyrene exposure.

**ANOVA table**	**SS**	**DF**	**MS**	**F (DFn, DFd)**	***p* value**
Interaction	551	3	184	F (3, 48) = 3	P = 0.0417
Row Factor	4	1	4	F (1, 48) = 0.06	P = 0.8134
Column Factor	730	3	243	F (3, 48) = 4	P = 0.0140
Residual	2981	48	62		

Table S2. Transcriptomic raw data of F0 adult male medaka testicular tissues with and without embryonic benzo[a]pyrene exposure.

Sample	Raw_Read_Number	Clean_Reads	Raw_Q20_rate	Total_Mapped	Multiple_Mapped	Uniquely_Mapped
Con1	46731618	43617064	97.76	39819779 (91.29%)	3501085 (8.79%)	36318694 (91.21%)
Con2	41820042	40157120	97.94	37494951 (93.37%)	3894696 (10.39%)	33600255 (89.61%)
Con3	43844346	40644452	97.59	37590237 (92.49%)	3409016 (9.07%)	34181221 (90.93%)
Con4	42243136	36985326	97.75	33838331 (91.49%)	2977702 (8.80%)	30860629 (91.20%)
2.5B[a]P1	45901414	42459884	97.73	39528868 (93.10%)	3576325 (9.05%)	35952543 (90.95%)
2.5B[a]P2	41653098	38606330	97.6	34999645 (90.66%)	3399064 (9.71%)	31600581 (90.29%)
2.5B[a]P3	41745736	38748954	97.91	35576272 (91.81%)	3486329 (9.80%)	32089943 (90.20%)
2.5B[a]P4	43271488	40160538	97.58	37079980 (92.33%)	3981829 (10.74%)	33098151 (89.26%)
80B[a]P1	47412732	43446770	97.87	39346548 (90.56%)	3372910 (8.57%)	35973638 (91.43%)
80B[a]P2	43408332	39062000	97.77	35948390 (92.03%)	2291374 (6.37%)	33657016 (93.63%)
80B[a]P3	43701846	40763396	97.81	36835261 (90.36%)	3018963 (8.20%)	33816298 (91.80%)
80B[a]P4	39802404	39317692	97.62	36151380 (91.95%)	3219965 (8.91%)	32931415 (91.09%)

Table S3. Significantly altered KEGG pathways in testes of matured medaka with embryonic benzo[a]pyrene exposure at 2.5 μg/L for 8 days.

**Pathways**	**ID**	***p* value**	**Up-DEGs**	**Down-DEGs**
Metabolic pathways	hsa01100	2.78E-08	ACSL5	ACOX3, PLCB1, BHMT, CHIT1, PRODH2, G6PC1, UGT2B17, ABAT, CYP8B1, UPB1, XDH, ACMSD
beta-Alanine metabolism	hsa00410	1.43E-05	-	UPB1, ACOX3, ABAT
Peroxisome	hsa04146	0.000195	ACSL5	-
Propanoate metabolism	hsa00640	0.001011	-	ACOX3, ABAT
Fatty acid degradation	hsa00071	0.001648	ACSL5	ACOX3
Adipocytokine signaling pathway	hsa04920	0.003876	ACSL5	G6PC1
Caffeine metabolism	hsa00232	0.007763	-	XDH
Inflammatory mediator regulation of TRP channels	hsa04750	0.007831	-	PLCB1, MAP2K6
Primary bile acid biosynthesis	hsa00120	0.023111	-	CYP8B1
Fatty acid biosynthesis	hsa00061	0.024379	ACSL5	-
Pantothenate and CoA biosynthesis	hsa00770	0.025646	-	UPB1
Rap1 signaling pathway	hsa04015	0.031067		PLCB1, MAP2K6
alpha-Linolenic acid metabolism	hsa00592	0.033213	-	ACOX3
Human cytomegalovirus infection	hsa05163	0.035197	-	PLCB1, MAP2K6
Biosynthesis of unsaturated fatty acids	hsa01040	0.035723	-	ACOX3

Ascorbate and aldarate metabolism	hsa00053	0.035723	-	UGT2B17
Butanoate metabolism	hsa00650	0.036975	-	ABAT
Galactose metabolism	hsa00052	0.040723	-	G6PC1
Pentose and glucuronate interconversions	hsa00040	0.044456	-	UGT2B17
Starch and sucrose metabolism	hsa00500	0.046937	-	G6PC1
Alanine, aspartate and glutamate metabolism	hsa00250	0.046937	-	ABAT
African trypanosomiasis	hsa05143	0.048175	-	PLCB1

Table S4. Significantly altered KEGG pathways in testes of matured medaka with embryonic benzo[a]pyrene exposure at 80 μg/L for 8 days.

**Pathways**	**ID**	***p* value**	**Up-DEGs**	**Down-DEGs**
Metabolic pathways	hsa01100	9.11E-14	STAR, AMPD3, MAT1A, PLCG1, PGAM2, ALPL, CYP2U1, AFMID, PLPP2, ADCY5, ACSS3, MGLL, CYP2R1, BDH2, HPD, GALNT14, PLA2G10, PFKFB1, AKR1D1, GLUL, WBP1, HAAO	CD38, PGM2L1, GAMT, G6PC1, CYP17A1, DMGDH, ABAT, PMM2, ALAD, CYP4F3, PANK2, BHMT, CTPS1, GK, TKTL2, CEL, DHRS3, IMPDH2, RRM2, PTS, ALDH3B2, ST3GAL1, UCK2, ALDH6A1, CYP1A1, CHAC1, PIPOX, AK2, CBS, ANPEP, HSD3B2, PNP, AHCY
ABC transporters	hsa02010	2.56E-06	ABCG4, ABCA1, CFTR, ABCA3	ABCC9, ABCB4, ABCC10
Transcriptional misregulation in cancer	hsa05202	4.67E-06	MLF1, PDGFA, ASPSCR1, ETV4, MMP9, BAIAP3	CEBPB, NR4A3, MEN1, PER2, NGFR, MYC
Pancreatic secretion	hsa04972	5.25E-06	ADCY5, PLA2G10, CFTR	CD38, CPA1, CEL, CPB1, CTRB1, PRSS2
Proteoglycans in cancer	hsa05205	1.08E-05	FZD3, IHH, EZR, MSN, PLCG1, ANK3, SMAD2, MAPK12, MMP9	MAP2K2, THBS1, MYC
Leukocyte transendothelial migration	hsa04670	1.43E-05	CLDN11, PTK2B, EZR, RAPGEF4, MSN, PLCG1, MAPK12, MMP9, VASP	-
Rap1 signaling pathway	hsa04015	1.49E-05	PDGFA, PDGFRA, ADCY5, PARD6G, RAPGEF4, PLCG1, MAPK12, VASP	THBS1, MAP2K2, NGFR, ANGPT4
Fat digestion and absorption	hsa04975	1.85E-05	PLPP2, PLA2G10, ABCA1	CEL, APOB, APOA1
Axon guidance	hsa04360	1.96E-05	NFATC2, FZD3, PARD6G, SEMA6B, PLCG1, SLIT2, TRPC4, PLXNA2, PAK5, SEMA7A	GDF7
Protein digestion and absorption	hsa04974	2.18E-05	SLC7A8, SLC3A2, COL18A1	CPA1, CPB1, CTRB1, COL6A2, PRSS2
Hepatitis B	hsa05161	0.0002195	STAT6, NFATC2, SMAD2, PTK2B,	MAP2K2, ATF4, MYC

			MAPK12, MMP9	
Th17 cell differentiation	hsa04659	0.0004203	STAT6, NFATC2, IL27RA, SMAD2, PLCG1, CD247, MAPK12	-
Phospholipase D signaling pathway	hsa04072	0.0005566	PDGFA, PDGFRA, PLPP2, ADCY5, PLCG1, RAPGEF4, PTK2B	MAP2K2
Cushing syndrome	hsa04934	0.0007424	ADCY5, FZD3, CACNA1D	CYP17A1, MEN1, MAP2K2, HSD3B2, ATF4
ECM-receptor interaction	hsa04512	0.0007872	GP1BB, ITGB8, ITGA7, LAMC1	COL6A2, THBS1
Pathways in cancer	hsa05200	0.0008779	PDGFA, PDGFRA, LAMC1, NFE2L2, STAT6, ADCY5, PPARD, SMAD2, PLCG1, FZD3, MMP9	MAP2K2, HLF, MYC, EPAS1, TXNRD3
RNA transport	hsa03013	0.0010912	EIF2B3, SAP18, EIF4EBP2	EEF1A2, KPNB1, EIF3D, PRMT5, EIF2S3
Protein processing in endoplasmic reticulum	hsa04141	0.0010912	NFE2L2, DERL1, FBXO2, WBP1	PDIA3, ATF4, SEC61A1, XBP1
Prion diseases	hsa05020	0.00113	LAMC1, C7	MAP2K2, C5
Hepatocellular carcinoma	hsa05225	0.001218	FZD3, NFE2L2, SMAD2, PLCG1	MAP2K2, MYC, SMARCA4, TXNRD3
Purine metabolism	hsa00230	0.0012478	ADCY5, STAR, AMPD3	AK2, IMPDH2, RRM2, PNP
Hematopoietic cell lineage	hsa04640	0.0014167	GP1BB	CR2, CD38, CD34, ANPEP, CD9
Cortisol synthesis and secretion	hsa04927	0.0014357	CACNA1D, ADCY5	CYP17A1, ATF4, HSD3B2
T cell receptor signaling pathway	hsa04660	0.0018917	NFATC2, PLCG1, CD247, MAPK12, PAK5	MAP2K2
Bladder cancer	hsa05219	0.0019364	MMP9	MAP2K2, MYC, THBS1
Human cytomegalovirus infection	hsa05163	0.0019655	ADCY5, NFATC2, PDGFRA, PTK2B, MAPK12	PDIA3, MAP2K2, ATF4, MYC
Biosynthesis of amino acids	hsa01230	0.0025914	PGAM2, GLUL	CBS, TKTL2, MAT1A

Complement and coagulation cascades	hsa04610	0.0032031	F10, C7	CR2, C5, FGB
Ovarian steroidogenesis	hsa04913	0.0035349	ADCY5	CYP17A1, CYP1A1, HSD3B2
MAPK signaling pathway	hsa04010	0.0035946	PDGFA, PDGFRA, CACNA1D, MAPK12, CACNG4	MAP2K2, NGFR, ATF4, MYC, ANGPT4
Vibrio cholerae infection	hsa05110	0.0037827	PLCG1, TJP1, CFTR	SEC61A1
Wnt signaling pathway	hsa04310	0.0038069	SFRP2, NFATC2, FZD3, PPARD, CBY1	SFRP5, MYC
Vitamin digestion and absorption	hsa04977	0.0038549	SLC52A3	APOB, APOA1
Platelet activation	hsa04611	0.0045364	ADCY5, GP1BB, BTK, MAPK12, VASP	FGB
Human immunodeficiency virus 1 infection	hsa05170	0.0048116	NFATC2, PLCG1, PTK2B, CD247, MAPK12, PAK5	PDIA3, MAP2K2
Gap junction	hsa04540	0.0049489	PDGFA, PDGFRA, TJP1, ADCY5	MAP2K2
PD-L1 expression and PD-1 checkpoint pathway in cancer	hsa05235	0.0051774	NFATC2, CD247, PLCG1, MAPK12	MAP2K2
Regulation of lipolysis in adipocytes	hsa04923	0.0051991	ADCY5, MGLL, LIPE	ABHD5
Relaxin signaling pathway	hsa04926	0.0056384	ADCY5, SMAD2, MAPK12, MMP9	MAP2K2, ATF4
Pyrimidine metabolism	hsa00240	0.0058528		PNP, UCK2, RRM2, CTPS1
Th1 and Th2 cell differentiation	hsa04658	0.0059076	STAT6, NFATC2, CD247, PLCG1, MAPK12	-
TGF-beta signaling pathway	hsa04350	0.0064329	SMAD2	NBL1, MYC, GDF7, THBS1
Lysine degradation	hsa00310	0.0065591	EZH1, PLOD1	TMLHE, PIPOX
VEGF signaling pathway	hsa04370	0.0065591	NFATC2, PLCG1, MAPK12	MAP2K2
Dilated cardiomyopathy	hsa05414	0.0069901	ITGB8, ITGA7, CACNA1D, CACNG4, ADCY5	-

Prostate cancer	hsa05215	0.007281	MMP9, PDGFA, PDGFRA	ATF4, MAP2K2
Glycerolipid metabolism	hsa00561	0.0073197	MGLL, PLPP2	GK, CEL
Aldosterone synthesis and secretion	hsa04925	0.0075802	CACNA1D, LIPE, ADCY5	HSD3B2, ATF4
Choline metabolism in cancer	hsa05231	0.0078878	PDGFA, PLCG1, PLPP2, PDGFRA	MAP2K2
Ras signaling pathway	hsa04014	0.0079823	PDGFA, PDGFRA, PLA2G10, PLCG1, PAK5	MAP2K2, NGFR, ANGPT4
Fructose and mannose metabolism	hsa00051	0.0087031	PFKFB1, PFKFB4	PMM2
Propanoate metabolism	hsa00640	0.0093894	ACSS3	ALDH6A1, ABAT
Acute myeloid leukemia	hsa05221	0.0094693	PPARD	PER2, MYC, MAP2K2
Adrenergic signaling in cardiomyocytes	hsa04261	0.0104173	ADCY5, CACNA1D, RAPGEF4, MAPK12, CACNG4	ATF4
Amphetamine addiction	hsa05031	0.0104324	PPP1R1B, CACNA1D, ADCY5	ATF4
Fc epsilon RI signaling pathway	hsa04664	0.0104324	PLCG1, MAPK12, BTK	MAP2K2
Renal cell carcinoma	hsa05211	0.0109368	PAK5	MAP2K2, EPAS1, HLF
Central carbon metabolism in cancer	hsa05230	0.0109368	PDGFRA, PGAM2	MAP2K2, MYC
MicroRNAs in cancer	hsa05206	0.0114173	PDGFA, PDGFRA, FZD3, EZR, PLCG1, MMP9	THBS1, MAP2K2, MYC
Prolactin signaling pathway	hsa04917	0.0114567	MAPK12	MAP2K2, SOCS3, CYP17A1
Bile secretion	hsa04976	0.0125435	ADCY5, CFTR	SLC22A7, ABCB4
TNF signaling pathway	hsa04668	0.0127073	MMP9, MAPK12	CEBPB, SOCS3, ATF4
Glioma	hsa05214	0.0142935	PDGFA, PLCG1, PDGFRA	MAP2K2
Gastric acid secretion	hsa04971	0.0142935	ADCY5, EZR, KCNK10, CFTR	
Jak-STAT signaling pathway	hsa04630	0.0150081	PDGFA, PDGFRA, STAT6, IL27RA	SOCS3, MYC

Arrhythmogenic right ventricular cardiomyopathy	hsa05412	0.0155419	ITGB8, CACNA1D, CACNG4, ITGA7	-
Neurotrophin signaling pathway	hsa04722	0.0159847	PLCG1, MAPK12	MAP2K2, NGFR, ATF4
Tryptophan metabolism	hsa00380	0.0160043	AFMID, HAAO	CYP1A1
EGFR tyrosine kinase inhibitor resistance	hsa01521	0.0168567	PDGFA, PLCG1, PDGFRA	MAP2K2
Yersinia infection	hsa05135	0.0170152	PLCG1, NFATC2, PTK2B, MAPK12	MAP2K2,
cGMP-PKG signaling pathway	hsa04022	0.0170976	ADCY5, NFATC2, CACNA1D, VASP	MAP2K2, ATF4
Human T-cell leukemia virus 1 infection	hsa05166	0.0182753	ADCY5, NFATC2, SMAD2	TBP, MAP2K2, ATF4, MYC
Thiamine metabolism	hsa00730	0.0187174	ALPL	AK2
B cell receptor signaling pathway	hsa04662	0.0189558	NFATC2, BTK	CR2, MAP2K2,
Human papillomavirus infection	hsa05165	0.0200233	FZD3, PARD6G, ITGB8, ITGA7, LAMC1	THBS1, MAP2K2, TBP, COL6A2
Phenylalanine metabolism	hsa00360	0.0207584	HPD	ALDH3B2
ErbB signaling pathway	hsa04012	0.0212098	PAK5, PLCG1	MYC, MAP2K2
Insulin secretion	hsa04911	0.0219961	RAPGEF4, CACNA1D, ADCY5	ATF4
Natural killer cell mediated cytotoxicity	hsa04650	0.0228274	NFATC2, CD247, PTK2B, PLCG1	MAP2K2
Dopaminergic synapse	hsa04728	0.0228274	PPP1R1B, CACNA1D, MAPK12, ADCY5	ATF4
FoxO signaling pathway	hsa04068	0.0234709	SMAD2, MAPK12, FOXO6	MAP2K2, G6PC1
Cocaine addiction	hsa05030	0.0234721	PPP1R1B, ADCY5	ATF4
GABAergic synapse	hsa04727	0.0244605	ADCY5, GLUL, CACNA1D	ABAT
Steroid biosynthesis	hsa00100	0.0251022	CYP2R1	CEL
Hypertrophic cardiomyopathy	hsa05410	0.0253176	ITGB8, CACNA1D, CACNG4, ITGA7	-
Amyotrophic lateral sclerosis	hsa05014	0.025897	DERL1, MAPK12	TOMM40

Fluid shear stress and atherosclerosis	hsa05418	0.0283035	PDGFA, ARHGEF2, NFE2L2, MAPK12, MMP9	-
Fc gamma R-mediated phagocytosis	hsa04666	0.0289251	ARPC1B, PLCG1, PLPP2, VASP	-
Signaling pathways regulating pluripotency of stem cells	hsa04550	0.0290414	FZD3, SMAD2, MAPK12	MAP2K2, MYC,
Endocytosis	hsa04144	0.0301101	PDGFRA, PARD6G, ARPC1B, SMAD2, ACAP3	IQSEC3, FOLR2
Pathogenic *Escherichia coli* infection	hsa05130	0.0311335	ARPC1B, ARHGEF2, EZR	-
Cell adhesion molecules	hsa04514	0.0337238	CLDN11, ITGB8, F3, CADM3	CD34
Epstein-Barr virus infection	hsa05169	0.0367339	CD247, MAPK12, BTK	CR2, PDIA3, MYC
mTOR signaling pathway	hsa04150	0.0397474	SLC3A2, FZD3, GRB10	MAP2K2, SLC38A9
Ribosome biogenesis in eukaryotes	hsa03008	0.0403464	-	HEATR1, NHP2, WDR43, DKC1
Parathyroid hormone synthesis, secretion and action	hsa04928	0.0414946	MMP17, PTHLH, ADCY5	ATF4
Folate biosynthesis	hsa00790	0.0428179	ALPL	PTS
Arachidonic acid metabolism	hsa00590	0.0431337	PLA2G10, CYP2U1	CYP4F3
Butanoate metabolism	hsa00650	0.0485267	BDH2	ABAT
Cholinergic synapse	hsa04725	0.0487694	KCNQ4, CACNA1D, ADCY5	ATF4
